# Interactions between the Cyclooxygenase Metabolic Pathway and the Renin-Angiotensin-Aldosterone Systems: Their Effect on Cardiovascular Risk, from Theory to the Clinical Practice

**DOI:** 10.1155/2018/7902081

**Published:** 2018-10-02

**Authors:** Jakub Gawrys, Karolina Gawrys, Ewa Szahidewicz-Krupska, Arkadiusz Derkacz, Jakub Mochol, Adrian Doroszko

**Affiliations:** Department of Internal Medicine, Occupational Diseases, Hypertension and Clinical Oncology, Wroclaw Medical University, Borowska 213, 50-556 Wroclaw, Poland

## Abstract

Coronary artery disease (CAD) and stroke are the most common and serious long-term complications of hypertension. Acetylsalicylic acid (ASA) significantly reduces their incidence and cardiovascular mortality. The RAAS activation plays an important role in pathogenesis of CVD, resulting in increased vascular resistance, proliferation of vascular-smooth-muscle-cells, and cardiac hypertrophy. Drugs acting on the renin-angiotensin-aldosterone system (RAAS) are demonstrated to reduce cardiovascular events in population with cardiovascular disease (CVD). The cyclooxygenase inhibitors limit the beneficial effect of RAAS-inhibitors, which in turn may be important in subjects with hypertension, CAD, and congestive heart failure. These observations apply to most of nonsteroidal anti-inflammatory drugs and ASA at high doses. Nevertheless, there is no strong evidence confirming presence of similar effects of cardioprotective ASA doses. The benefit of combined therapy with low-doses of ASA is—in some cases—significantly higher than that of monotherapy. So far, the significance of ASA in optimizing the pharmacotherapy remains not fully established. A better understanding of its influence on the particular CVD should contribute to more precise identification of patients in whom benefits of ASA outweigh the complication risk. This brief review summarizes the data regarding usefulness and safety of the ASA combination with drugs acting directly on the RAAS.

## 1. Introduction

Activation of the renin-angiotensin-aldosterone system (RAAS) plays an important role in the pathogenesis of cardiovascular disorders, resulting in increased vascular resistance, excessive fluid retention, increased proliferation of vascular-smooth-muscle-cells, and cardiac hypertrophy. Inhibition of the bradykinin degradation, catalysed by the angiotensin converting enzyme (ACE), results in increased prostacyclin release and constitutes one of the additional mechanisms of the ACE inhibitors (ACE-I) antihypertensive effect. Bradykinin, physiologically degraded by ACE, is accumulated resulting in vasodilatation, which is partially dependent on the prostanoid metabolic pathways, nitric oxide, and endothelium-derived hyperpolarizing factor (EDHF). The cyclooxygenase (COX) inhibitors affect the above-mentioned mechanisms and may limit some beneficial effects of ACE-I. This in turn might be important in patients with hypertension, coronary artery disease (CAD), and congestive heart failure (CHF). These observations apply to most of nonsteroidal anti-inflammatory drugs (NSAIDs) and aspirin (acetylsalicylic acid, ASA) at high doses. Nevertheless, there is no strong evidence, based on the long-term prospective studies, confirming the presence of similar effects of ASA when administered in small, cardioprotective doses [1].

## 2. Renin-Angiotensin-Aldosterone System and the Cyclooxygenase Metabolic Pathway

Recent studies have clearly demonstrated the role of aldosterone in the pathogenesis of systemic inflammation, endothelial dysfunction, and fibrosis [2, 3]. It has also been shown that the effects of mineralocorticoids are present not only in the kidneys and that they are synthesized also outside the adrenal cortex [4]. Aldosterone acts on the cardiovascular system and also on the mechanisms independent of blood pressure regulation. It has been proven that the pharmacological blockade of its receptor exerts protective effects on the cardiovascular system, even while maintaining a constant value of blood pressure [5, 6].

A large amount of data indicates the participation of COX-dependent pathways in the pathogenesis of endothelial dysfunction induced by aldosterone. Its development is also possible in the normotension, and it is suppressed by both aldosterone receptor blockade and the COX inhibition [7]. Further analysis of the connections between these elements has shown that the thromboxane (TX) receptor blockade reduces the severity of endothelial dysfunction induced by aldosterone, but similar results were not observed for the inhibition of its synthesis. The increased production of TXA2 by vessels with aldosterone-induced endothelial dysfunction has also been shown, which—in combination with the previously cited observations—would indicate the TXA2 as a key mediator in development of aldosterone-mediated endothelial dysfunction. However, taking into account the fact that the thromboxane synthesis inhibitors do not inhibit the vascular reaction to acetylcholine, it is postulated that the TX does not have a significant pathogenic role, which does have other prostanoids acting through the TX receptor [8–11].

Prostacyclin (PGI), apart from the recognized vasodilatory action, can pave the pressor effect* via* the TX receptors, which takes place, for instance, in the aorta, where PGI seems to have, next to the PGH2, the largest share in the activity of EDCF [12, 13]. Moreover, it has been shown that in certain situations the inhibition of prostacyclin synthesis can improve the diastolic reactivity of the vessel.

According to Blanco-Rivero [7], exposure to aldosterone induces endothelial dysfunction* via* activation of COX-2 both in case of initially normal blood pressure as well as in experimental models of hypertension. In the second case, the PGI2 appears to play a key role, but under normotensive conditions it is assigned to different prostanoids (PGE2, PGF2).

Another interesting aspect of the mineralocorticoids activity is their different effect on the endothelium, dependent on the exposure duration. In the short term, a vasodilatory reaction is observed which is explained by activation of NO-dependent pathways, as well as an effect of vasodilatory acting prostanoids. However, a clear tendency to vasoconstriction, which can be explained by the development of inflammatory processes, is observed in the long-term exposure and it exemplifies the negative consequences of the chronic RAAS hyperactivity.

## 3. Endothelial Function

Endothelial dysfunction, present in almost all of the cardiovascular disorders (CVD), develops as a result of nitric oxide and other vasodilators' deficit, with superiority of strong vasoconstrictive factors of proinflammatory action, such as endothelin I, angiotensin II, or some of the prostanoids [26, 27].

A widely understood imbalance among the whole group of factors with pressor activity [TXA2, isoprostanoids, 20-Hydroxyeicosatetraenoic acid, superoxide anion, H_2_O_2_, endothelin-1, angiotensin II, and UTP] and vasodilators [adenosine, prostacyclin (PGI2) and nitrogen oxide (NO), epoxyeicosatrienoic acids (EETS), and C-natriuretic peptide] may play a potential role in vascular tone regulation. The cooperation of endothelial cells with vascular-wall-smooth-muscle-cells is possible through the gap-junctions. It is manifested, i.e., by conduction of electric potential in response to EDHF or by transfer of small ions [28].

The beneficial effect of ASA in CVD is often attributed to its antiaggregative properties. The influence of ASA and other NSAIDs on the pro- and anti-inflammatory activity of endothelium is almost completely ignored. Retention of sodium, exacerbated by COX, can be compensated by other mechanisms to prevent the blood pressure increase. On the other hand, prostaglandin E2 and prostacyclin I2 are the determinants of renin secretion. Interfering with their metabolism may contribute to development of hypertension, especially in the course of renal artery stenosis. Fujino et al. [29] have shown that the deficiency of prostacyclin (PGI2) receptor prevents the development of renovascular hypertension.

Common transduction system beginning from the second messenger (cAMP) for prostacyclin, PGE2, EP2, and EP4 receptors may suggest the occurrence of similar effects after functional blockade of the EP2 and EP4 signalling [30]. Nevertheless, the study by Fujino et al. does not confirm this hypothesis, indicating clearly the key role of PGI2 and its receptors in the development of renovascular hypertension. The exact mechanism of this interaction remains unknown. A lot of data indicates the possibility of modulating the local activity of the adrenergic system to be responsible for the renin release regulation from the juxtaglomerular apparatus. Another hypothesis, opposing the results of Fujino et al., assumes the existence of an interaction at the level of dense dot stimulating the release of renin in response to a reduced chloride content in the urine. Decreased expression of the second symporter Na, K/2Cl (2NKCC2), leads to activation of COX-2 and increased synthesis of PGE2 followed by EP2 receptor activation and in consequence by releasing a greater amount of renin [31–33].

To summarize, the paradoxical duality of the prostanoids' influence on the regulation of blood pressure should be noticed. On one hand, there is an activation of the RAAS by COX-2-mediated PGI2 and, on the other hand, vasodilatory effect of PGI2 on blood vessels is exerted by endothelium and intensification of natriuresis. Under physiological condition, the state of dynamic balance between these opposing mechanisms is determined, and it is significantly altered in pathology. Further understanding of these complex interactions should allow the selection of two different groups of patients: the first one where the use of NSAIDs exerts adverse hypertensive effects and the second one with significant benefits of pharmacological blockade of the cyclooxygenase activity.

## 4. Platelet Function

Platelets have a well-defined role in coronary artery thrombosis and in other cardiovascular disorders, including diabetes mellitus, stroke, and peripheral vascular disease.

ASA acts by irreversible acetylation of the COX1 serine residue which blocks the access of substrate, arachidonic acid, to the catalytic site of the enzyme limiting the thromboxane A2 synthesis leading to the inhibition of platelet aggregation [34–37]. Aspirin can also acetylate other proteins, like albumins or haemoglobin, but acetylation of the COX1 enzyme occurs at micromolar concentrations, which suggests the specificity of this reaction. The impact of other NSAIDs on platelet function also has been investigated. None of them irreversibly inhibited COX1 due to another mechanism of drug attachment to the enzyme. Some of the NSAIDs may interact with ASA during their concomitant administration. It has been found that ibuprofen and its derivatives prevent the access of aspirin molecule to the acetylation site and thus prevent the irreversible inactivation of this enzyme. This theory was confirmed in Catella-Lawson et al. study where the administration of ibuprofen before aspirin decreased the amount of inactivated platelets. This observation made at the molecular level has significant clinical implications because the aggregation occurs only in 85-90% of platelets. Theoretically, this interaction can be circumvented by administering ASA before ibuprofen, which was shown to be effective [36]. There were no similar interactions with the administration of ASA and rofecoxib (affinity for COX2) as well as diclofenac and acetaminophen (weak affinity for COX1) [36].

Angiotensin II contributes to increased platelet aggregation by activating the G protein-coupled receptor. This increases the Ca^2+^ concentration in platelets, which promotes fibrinogen receptor expression, increases thromboxane A2 production, and—in consequence—causes aggregation. ACE-I should thus inhibit platelet aggregation. Some studies confirm this by use of captopril ex vivo [38], but other authors obtained opposite results [39]. A study by Moser et al. [40], in which the impact of captopril, enalapril, and fosinopril on platelet functioning in hypertensive patients was investigated, showed only minimal impact of these drugs on the platelets aggregation, probably due to their antihypertensive action. However, the impact on thromboxane A2 formation varied depending on the substance used. Administration of captopril inhibits the formation of TXA2 but only for a short period of time. Enalapril does not affect its concentration, whereas fosinopril blocks the formation of thromboxane A2 probably through direct antagonist effect on TXA2 synthase activity. This may indicate the utility of fosinopril in limiting platelet activation.

Angiotensin receptor blockers, despite their ACE-I-like action, have additional properties affecting platelet function. One of them, irbesartan, reduces the concentration of TXA2 similarly to the above-mentioned fosinopril. The exact mechanism of this action is unknown, but it is suspected that it could act directly or by its metabolites on the TXA2 receptor [41]. Furthermore, losartan causes the release of nitric oxide from platelets and endothelial cells which activates the phosphorylation of TXA2/PGH2 receptors and inhibits intraplatelet TXA2 pathway [42].

Aldosterone antagonists also affect the function of platelets. Schafer et al. analyzed the impact of eplerenone on vascular function and platelet activation demonstrating reduced platelet aggregation following its administration in diabetic rats. This is accompanied by reduced production of superoxides and elevated nitric oxide bioavailability. Similar observations were done with spironolactone [43].

## 5. RAAS in Oxidative Stress, Aging, and Inflammation

Chronic oxidative stress is an important mediator in the development of hypertension. It has been shown that the superoxide dismutase (SOD) activating agents lead to the inhibition of endothelial apoptosis and may prevent its dysfunction [44, 45].

Pharmacological blocking of endothelin receptors in experimental models of diabetes leads to reduction of the NADPH oxidase expression and consequently significant reduction in oxidative stress [46, 47]. A similar effect has also been shown for drugs inhibiting the RAAS [48, 49].

Paradoxically, hypertension induced in animal models is characterized by increased activity of NO-mediated pathways in response to Ach stimulation, with relatively constant basic NO-release. It is considered as a compensatory mechanism, which is relatively rapidly downregulated [50, 51].

Aging also comes with a decrease in the EDHF expression, which results in reduced vasodilatory reactivity in NO-independent mechanisms [52, 53]. This effect may be partially reversed by the treatment with candesartan, most likely due to upregulation of the gap-junctions between endothelium and vascular-smooth-muscle-cells [54], and it is also influenced by the calcium channel blockers (CCBs), selective estrogenic receptor modulators (SERMs), or vasopeptidase inhibitors [55–57].

A study by Mukai et al. [58] evaluating effect of the RAAS pathway blockade on reducing the severity of age-related endothelial dysfunction associated in rats showed that long-term suppression of the RAAS leads to a significant reduction of endothelial dysfunction by reducing the excessive synthesis of COX2 products and superoxide anion.

Interesting data on chronopharmacological activity profile of acetylsalicylic acid is provided by Hermida et al. [59–62]. The results of most of them clearly indicate its hypotensive action when the drug was administered in the evening, which is not observed while taking it in the morning. The observed daily variability of ASA pharmacological profile can be explained by several mechanisms. One of them is the increased clearance of ASA after administration in the morning [63]. A potential mechanism that could explain this phenomenon is the effect of ASA on the thromboxane and prostacyclin synthesis, also presenting the circadian variations [64–66].

Donors of sulfhydryl groups (e.g., N-acetylcysteine), through the antioxidant properties, can potentiate the hypotensive effects of drugs via the nitric oxide dependent mechanisms, which is also observed in the case of ACE-I [67].

Endothelial and insulin signalling pathways have crosstalk between each other and therefore the relationship between endothelial function and insulin metabolism is very important. Insulin resistance impairs vascular reactivity and increases cardiovascular risk. Involvement of insulin resistance and endothelial dysfunction in pathological disorders contributes to impairment in the NO-dependent vasodilatation, cellular glucose uptake, enhancement in oxidative stress and inflammation (e.g., through increased synthesis of prostanoids and ROS as side products), hyperactivation of the RAAS, and increased generation of leading to atherosclerosis [68]. Regular aerobic physical activity seems to restore the homeostasis resulting in endothelial function recovery [69].

## 6. ASA and ACE-I: Their Pleiotropic Action

**Figure 1:**
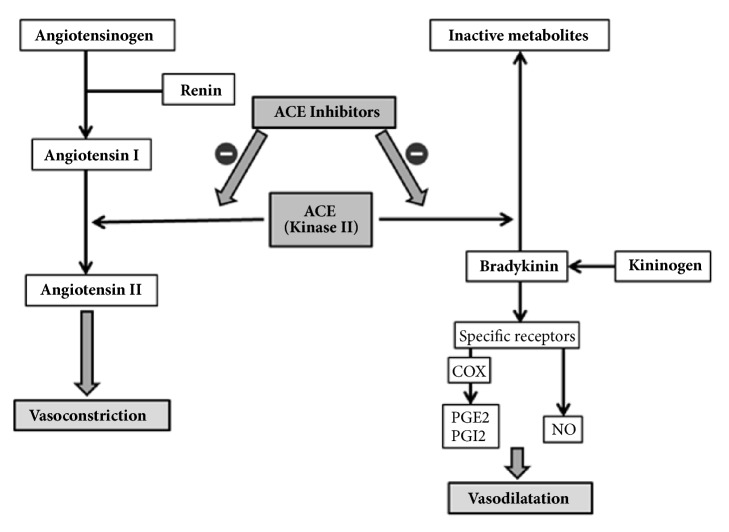
Theoretical background for an interaction between cyclooxygenase and the renin-angiotensin-aldosterone systems.

One clinical trial has shown the beneficial effect of aspirin on endothelial function and blood pressure control in hypertensive patients receiving statin. There were no similar results in a group without hypolipemic treatment, which may indicate the presence of synergism promoting pleiotropic effects of statins. Increase in the NO-bioavailability by ASA can contribute to the improvement of endothelial vasodilatory function and, as a result, a better control of blood pressure [70, 71]. On the other hand, inhibition of COX activity can reduce the beneficial effect of ACE inhibitors on endothelial vasodilatory function (Figure 1) [72].

In a study on diabetic patients with albuminuria it has been shown that addition of aspirin (at a dose of 300mg/24h) to enalapril does not abolish its nephroprotective properties because it does not lead to a reincrease of proteinuria previously reduced by the enalapril [73]. In one of the studies it has been shown that the interaction between aspirin and ACE inhibitors is observed at a dose of 500mg/24h ASA and does not occur when the daily dose of aspirin is 100 mg [20].

The results of some experimental studies have shown that the positive effect of ACE inhibitors on the postinfarction cardiac remodeling is mediated by bradykinin. Cardioprotective effect of ACE inhibitors by antioxidant activity is an effect of the activation of bradykinin receptor B2 and prostaglandin synthesis. This, in turn, could explain the reduction of this effect by COX inhibitors. Meune et al. demonstrated a dose-dependent negative effect of aspirin on the function of vascular wall in patients with heart failure treated with ACE inhibitor [74].

The blockade of cyclooxygenase may be particularly unfavorable in patients with heart failure treated with ACE-I, when limiting the prostanoid dependent activity of bradykinin causes an increase in peripheral resistance, a decrease in organ perfusion, and cardiac output. A different interaction profile is notable, depending on the selectivity of COX inhibitors—antagonism is greater with the increase of COX-1-selectivity. It may explain the clear pharmacodynamic antagonism observed between relatively more COX-1 selective indomethacin and ACE-I and in the case of ASA—a compound with comparable inhibitory activity on both isoforms of cyclooxygenase. The observed interactions are considerably poorer, especially for low ASA doses, when this affinity for tissue isoform of COX-1 is relatively smaller compared to the enzyme present in the platelets. Interestingly, in young healthy subjects, the presence of poor platelet responsiveness to ASA may be associated with development of endothelial dysfunction [75] and the common link could be the platelet carbonic anhydrase II [76].

Bhagat et al. showed that high doses of aspirin (i.e., 1000 mg/24h) inhibit the vasodilatation in response to arachidonate, which was absent when small doses of ASA were applied. However, in a few other studies conducted on patients with congestive heart failure (CHF), it was demonstrated that 75-150mg of ASA is capable of inhibiting the arachidonate-dependent vasodilatation. This observation can be explained by the greater efficacy of ASA in inhibiting the synthesis of prostacyclin in this group of patients [77].

## 7. Clinical Evidence on Interaction of ASA with ACE-I, Angiotensin Receptor Blockers (ARB), and Mineralocorticoid-Receptor-Antagonists (MRA)

### 7.1. Coronary Artery Disease (CAD)

Safety of the interaction between aspirin and ACE-I in the treatment of CAD has been the subject of numerous studies, but most of the data come from retrospective analyses. The GISSI-3 study [78] did not show the negative consequences of combining aspirin with ACE inhibitors (mortality in the placebo group and ACE-I without ASA, respectively, 13 and 10.8% vs. 6 and 5.4%, respectively, in the groups receiving aspirin concomitantly), and in the coexistence of diabetes combination of ASA and ACE-I led to a partial reduction of beneficial effects of ACE-I but still implied a significant reduction in mortality (the use of ACE-I reduced the mortality rate from 10.1 to 7.7% in the group previously treated with ASA and, respectively, from 24.7 to 13.5% in the group without ASA). The cohort study involving patients with CAD does not confirm the presence of pharmacodynamic antagonism between aspirin and ACE-I. Therefore the authors postulate the coadministration of these drugs in this group of patients. The results of the GISSI-3 show that concomitant use of aspirin and ACE-I is associated with a significant reduction in mortality in the acute phase of a heart attack. The CONSENSUS II study [14] demonstrated that the 6-month mortality in the patients with acute myocardial infarction and receiving concomitantly ACE inhibitors and aspirin is significantly higher, compared to patients treated with only one of these drugs. The CATS study (captopril and low-dose ASA) did not confirm this observation [15].

Administration of aspirin in combination with ARB in patients with CAD was examined by Levy et al. on the population from OPTIMIZE-HF study. The results showed no statistically significant side effects of coadministration of drugs from these two groups in a 60- and 90-day long period following discharge from the hospital [20]. In the case of coadministration of ASA and MRA (spironolactone and eplerenone), the addition of eplerenone to optimal medical therapy reduces morbidity and mortality among patients with acute myocardial infarction complicated by left ventricular dysfunction and heart failure. In the studied group, 89% of patients were given aspirin, as a part of optimal treatment [79, 80]. Additionally, the EPHESUS trial showed that, especially in patients with previous myocardial infarction, the early (3-7 days) addition of eplerenone to the optimal treatment significantly improved their prognosis [24, 81].

### 7.2. Congestive Heart Failure (CHF)

There is also some evidence for the positive interaction between aspirin and ACE-I leading to inhibition of thromboxane synthesis (in the case of ACE-I by blocking the synthesis of ATII) in patients with CHF. Furthermore, bradykinin is a potential factor releasing norepinephrine in synapses of the sympathetic nervous system. In this case, the use of aspirin can possibly decrease the adverse consequences of sympathetic nervous system stimulation, as one of the mechanisms involved in noradrenaline release in the myocardial sympathetic system is activation pathway mediated by prostanoids. However, the results of the small study conducted on 40 patients with an acutely decompensated CHF do not confirm the existence of such a relationship—the addition of ASA to ACE-I resulted in the deterioration of hemodynamic parameters and abrogated the beneficial effects of ACE-I [25, 82–86]. A study by Pedone et al. did not reveal the existence of a negative interaction between aspirin and ACE-I in a group of elderly subjects with CHF. The results of the SOLVD study are in opposition to the ones referenced above [16, 87]. Considering the mechanism of ASA action, it has been postulated that the inhibition of prostacyclin synthesis by greater doses of aspirin reduces the beneficial effect of ACE-I. The SOLVD study demonstrated that the addition of aspirin in patients treated with ACE inhibitors reduces the clinical benefit while adding the ACE-I reduces the beneficial effect of aspirin on the survival. Data from the WASH (Warfarin/Aspirin in Heart Failure) and WATCH (Warfarin and Antiplatelet Therapy in Chronic Heart Failure) studies showed a clear increase in hospitalizations for CHF in the group randomized to receive aspirin, thus indicating no benefit of ASA adding to the CHF therapy [17].

The OPTIMIZE-HF study demonstrates the absence of side effects of combining aspirin with ARB in both ischemic and nonischemic CHF. Similar conclusions were postulated by Chan Su Min et al., who studied the population of the CHARM trial, where no negative effect of candesartan supply with ASA on mortality in patients with chronic heart failure was shown [18]. Coadministration of ASA and the aldosterone antagonists reduce the risk of death in both the ischemic and nonischemic CHF [76].

### 7.3. Arterial Hypertension (HTN)

Another noteworthy issue is the significance of the aspirin and ACE-I interaction in the treatment of hypertension. Some meta-analyses indicate the possibility of an increase in blood pressure (RR) during the administration of selective COX-2 inhibitors. However, the observed changes tend to be too small to cause a significant increase in the relative risk [21], and in the case of ASA they are observed only for high doses [88]. In the study by Doroszko et al. it has been demonstrated that endothelial dysfunction in subjects with essential hypertension results not from decreased NO synthesis, but also from its increased degradation by reactive oxygen species (ROS) produced in the course of prostanoids' biosynthesis. Hence, limiting the activity of the arachidonic acid cascade could limit the impaired endothelial vasodilative reactivity in these patients [89].

In a study conducted by Guazzi et al. [1], it is demonstrated that low-doses of aspirin do not significantly affect the RR control in hypertensive patients treated with ACE inhibitor and the dose of 300 mg significantly reduces the antihypertensive effect of ACE inhibitors, but only in some patients. This subpopulation was characterized by a significantly higher incidence of severe hypertension and increased baseline activity of the RAA system. However, other studies do not confirm these observations, while highlighting the significant reduction in cardiovascular events in HTN patients taking ASA, which was reflected in the results of the HOT study [90]. Inhibiting of the renal prostaglandin production and decreased NO-bioavailability in kidneys by the NSAIDs and ASA at high doses may result in development of resistant hypertension [19].

An interesting study to verify the existence of the ACE-I class effect in the effect on the prostanoids' synthesis was conducted by Rodriguez-Garcia et al. The authors compared the effect of captopril, enalapril, ramipril, and fosinopril on the reduction of RR and the daily urinary excretion of the prostanoid pathway final metabolites (6-oxo-PGF-1 alpha and TXB2). Their study also included healthy subjects who did not receive any medications. A significantly lower baseline synthesis of prostacyclin in the study group was shown, and the hypotensive action of ACE-I, secondary to excessive synthesis of PGI, revealed being a class effect [91, 92]. Another small trial showed no reduction of the hypotensive effects of enalapril after administration of 150mg/d ASA [93]. Meune et al. conducted a study which was aiming at determining the minimal dose of ASA capable of limiting the beneficial effects of ACE inhibitors, noting a large interindividual variability in the dose of ASA, which is explained by the different pathogenesis of neurohormonal dysregulation in the vascular wall tension [73].

Proteins whose expression level is altered according to the RR values may be considered as markers of the blood pressure control. Interestingly, the induction of hypertension by an angiotensin II supply in an animal model accelerated aging of platelets and increased oxidative stress. It has been demonstrated that the treatment with angiotensin II receptor blockers increases the antioxidant status of platelets and makes them less susceptible to spontaneous activation in response to agonists including arachidonic acid [94].

There are many studies that describe the interaction of aspirin with ACE inhibitors. Much less data confirms the effects of coadministration of ASA and ARB. One of the studies, based on a LIFE (Losartan Intervention For Endpoint reduction) trial, indicates the positive effects of coadministration of drugs on reducing the frequency of MI and other endpoints in patients with hypertension [95]. In addition, the study conducted by Nawarskas et al. showed no differences in the values of mean as well as systolic and diastolic blood pressure during the treatment with only losartan and in its combination with a low (81mg/day) and high (325mg/day) dose of aspirin. The same researchers received similar results when replacing losartan with enalapril [22]. The data summarizing the papers on the outcomes of combined use of ASA and ACE-I, ARB, and MRA in coronary artery disease, congestive heart failure, and hypertension is summarized in Table 1.

## 8. Conclusions

Prospective clinical studies with hard endpoints revealed no clinically significant negative interaction between ASA at cardioprotective doses and ACE-I, ARB, or MRA. However, it has been shown that the combination of these groups of drugs provides much greater clinical benefits than using them separately. According to some authors, obtaining a synergistic effect and thus greater reduction in mortality can be achieved by the addition to the ACE-I therapy an antiplatelet drug with another, COX-independent mechanism of action [23, 96–99].

## Figures and Tables

**Table 1:** Data on the outcomes of combined use of ASA and ACE-I, ARB, and MRA in CAD, CHF, and HTN.

	**Coronary Artery Disease**	**Congestive Heart Failure**	**Hypertension**
**ASA + ACE-I**	Depends on trial: No side effects and reduction in mortality (n=19394) [14] vs. increase in mortality (n=6090) [15]	Reduction of clinical benefit (n=2569) [16, 17], increased number of hospitalizations (n=279 and n=1587) [18]	Dose-dependent: low doses of ASA do not affect RR; high doses (>300mg/d) may result in development of resistant hypertension (n=52) [1]. Reduction in cardiovascular events (n=18790) [19]

**ASA + ARB**	No side effects (n=41267) [20]	No side effects (n=41267 and n=4576) [20, 21]	Reduction in mortality (n=9193) [22], no changes in RR values (n=17) [23]

**ASA + MRA**	Reduction in mortality (n=6642) [24, 25]	Reduction in mortality (n=6642) [24, 25]	No changes in RR values, no data regarding mortality (n=17) [23]
